# Associations between diabetes status and grip strength trajectory sub-groups in adulthood: findings from over 16 years of follow-up in the MRC National Survey of Health and Development

**DOI:** 10.1186/s12877-023-03871-9

**Published:** 2023-04-04

**Authors:** T. Norris, W. Johnson, R. Cooper, S. M. Pinto Pereira

**Affiliations:** 1 Institute of Sport, Exercise and Health, Division of Surgery & Interventional Science, University College London, London, UK; 2 School of Sport, Exercise and Health Sciences, Loughborough University, Loughborough, UK; 3 AGE Research Group, Translational and Clinical Research Institute, Faculty of Medical Sciences, Newcastle University, Newcastle, UK; 4 NIHR Newcastle Biomedical Research Centre, Newcastle University and Newcastle upon Tyne NHS Foundation Trust, Newcastle, UK

**Keywords:** Ageing, Strength, Epidemiology, Trajectories, Diabetes

## Abstract

**Background:**

Cross-sectional studies suggest a relationship between diabetes status and weaker grip strength (GS) in adulthood and limited evidence from longitudinal studies has focussed on the association with average change in GS. We aimed to investigate whether diabetes status was related to membership of distinct GS trajectories in mid-to-late adulthood in 2,263 participants in the Medical Research Council National Survey of Health and Development.

**Methods:**

Grip strength (kg) was measured at 53, 60–64 and 69 years. Pre-/diabetes was defined at 53 years based on HbA1c > 5.6% and/or doctor-diagnosis of diabetes. Sex-specific latent class trajectory models were developed and multinomial logistic regression was used to investigate the association between pre-/diabetes status and membership into GS trajectory classes.

**Results:**

For both males and females, a 3-class solution (‘High’, ‘Intermediate’, ‘Low’) provided the best representation of the GS data and the most plausible solution. There was no evidence that pre-/diabetes status was associated with class membership in either sex: e.g., adjusted odds ratios of being in the ‘Low’ class (vs. ‘High’) for males with pre-/diabetes (vs. no-diabetes) was 1.07 (95% CI:0.45,2.55).

**Conclusion:**

Using a flexible data-driven approach to identify GS trajectories between 53 and 69 years, we observed three distinct GS trajectories, all declining, in both sexes. There was no association between pre-/diabetes status at 53 years and membership into these GS trajectories. Understanding the diabetes status―GS trajectories association is vital to ascertain the consequences that projected increases in pre-/diabetes prevalence’s are likely to have.

**Supplementary Information:**

The online version contains supplementary material available at 10.1186/s12877-023-03871-9.

## Background

Muscle weakness is associated with mobility disability [1] and a loss of independence; its prevalence increases with age, and it is a key component of age-related conditions such as sarcopenia [2] and frailty [3]. In light of an ageing population [4], muscle weakness is now recognised as a major public health concern with growing awareness of its personal, family- and societal-level impacts and its important contribution to the global burden of disease and disability [2].

Grip strength (GS), commonly used to identify muscle weakness, peaks in early adulthood and is maintained through mid-life before declining [5]. However, between-individual variation exists: e.g., there is heterogeneity in the rate of decline in strength in later adulthood [6, 7], with faster rates of decline associated with increased rates of mortality [7, 8]. Therefore, understanding variability in GS trajectories at older ages and identifying factors associated with such variability may help identify individuals at increased risk of low strength, subsequent morbidity, and premature mortality.

Knowledge of factors associated with GS in later adulthood has predominantly been based on cross-sectional studies with GS measured at a single timepoint. However, in older adults, GS at any one age is a function of peak GS achieved, age of decline onset and rate of GS decline [9]. With only a single GS measurement, distinguishing between these is not possible. More recently, studies with longitudinal GS data in later adulthood have emerged, attempting to summarise the GS trajectory [10, 11] and identify associated factors from earlier in the life-course [12, 13]. However, these studies assume a single ‘average’ GS trajectory around which individuals vary, overlooking the possible existence of sub-populations that have different timings and/or rates of decline in GS. The latter is more realistic, given evidence supporting the existence of heterogeneous healthy ageing phenotypes [14], including GS decline [6, 7].

Evidence for the existence of sub-populations of GS trajectories in adulthood has emerged from analyses in the Medical Research Council (MRC) National Survey of Health and Development (NSHD). Cooper and colleagues categorised individuals into four groups, ‘decline’, ‘stable high’, ‘stable low’ and a ‘reference’ group, based on changes in their sex-specific standard deviation GS scores between 53 and 60–64 years(y) [15, 16]. While different risk factor profiles were observed between these four groups [15], the availability of only two GS measurements meant that more complex modelling techniques which can more accurately detect trajectory sub-populations could not be used. Subsequently, a further wave of follow-up occurred in the NSHD at 69y. Using this additional GS measurement, Dercon and colleagues [17] applied more complex modelling to demonstrate the existence of latent GS trajectory sub-populations. These models combined sexes (despite known sex-differences in GS [5]) and participants with only a single GS measurement were included (potentially resulting in a misrepresentation of the underlying GS trajectories). As such, a re-analysis is warranted to determine whether similar sex-specific sub-populations are evident when limited to individuals with repeated GS data.

If distinct sub-populations of GS trajectory are apparent in mid-late adulthood, this would allow interrogation into how these groups differ with respect to modifiable factors, providing opportunities for developing interventions to potentially delay/minimise the rate of decline in strength with age. One such modifiable factor which may differ across GS-trajectory sub-populations is diabetes status. Cross-sectional studies demonstrate associations between diabetes status (including pre-diabetes markers and glucose levels) with lower GS [18, 19]. However, in addition to being unable to rule out reverse causality, cross-sectional studies do not enable investigation into whether diabetes status is associated with changes in GS over time. Recently, the relationship between baseline glucose levels and GS trajectories over seven years (n = 3 GS measurements, mean age at baseline = 71y) was investigated [20]: those in the highest quartile of fasting plasma glucose (FPG) had persistently weaker GS trajectories than those with lower FPG. However, because a single ‘average’ GS trajectory was assumed, it was not possible to elucidate whether FPG was associated with distinct GS trajectory sub-populations.

To address current knowledge gaps and methodological limitations of existing studies, we aimed to investigate whether diabetes status was related to membership into distinct trajectories of GS in mid-late adulthood using data collected in a large, nationally representative birth cohort.

## Methods

The MRC NSHD is a birth cohort which has been described in detail [21–23]. Briefly, it is a stratified sample of all births in one week in March 1946 in mainland Britain, comprising 5,362 individuals followed, so far, to age 69y. The only inclusion criterion for our study was the availability of at least two GS measurements collected at ages 53y (1999), 60-64y (2006–2010) and 69y (2015). This resulted in an analysis sample of 2,263 participants. Of the remaining 3,099 in the original cohort, 855 died (n = 214 before 53y) and 2,244 provided fewer than two measures of GS (See Table S1 for differences in the included versus excluded sample).

Ethical approval was provided including by the: North Thames Multi-Centre Research Ethics Committee (REC) (1999); Central Manchester REC (07/H1008/168) and Scottish A REC 2006–2010; National Research Ethics Service Queen Square REC (14/LO/1073) and Scotland A REC (14/SS/1009) (2015). Participants gave written informed consent [21–23].

### Exposure: pre-/diabetes

We derived a dichotomous ‘pre-/diabetes’ variable using data collected at 53y. At 53y non-fasting blood samples were analysed for HbA1c with the Tosoh A1C 2.2 Plus Analyzer (Tosoh, Tokyo, Japan). Pre-/diabetes was defined as HbA1c > 5.6% [24] and/or self-report of doctor-diagnosis of diabetes (by 53y).

### Outcome: serial grip strength

During nurse assessments at 53 and 60–64y, GS was measured in kilograms isometrically using a Nottingham electronic handgrip dynamometer; during a nurse home visit at 69y, a Jamar Plus + Digital Hand dynamometer was used. At each age, the same standardised protocols were applied. We use the maximum of the first four measures (two in each hand). (See Tables S2 and S3 for distribution of GS and age at each measurement sweep.)

### Covariates

Potential confounders of the pre-/diabetes―GS association were identified a-priori and included in the directed acyclic graph (Figure S1). Occupational class at 53y was classified into six groups from professional to unskilled; educational attainment at 26y was classified into 4 groups from no education to degree level; self-reported leisure time physical activity in last four weeks, smoking status and diagnoses of cancer and ‘severe respiratory symptoms’ were classified as binary variables (see Table 1); height, waist and hip circumferences were measured at 53y following standard protocols. Waist-hip ratio (WHR) was derived.

**Table 1:** Descriptive statistics for each grip strength trajectory latent class (n = 2263)

Variable (reporting age (y))	Missing (n,%)	*Total sample*	*High grip strength*	*Intermediate grip strength*	*Low grip strength*
	Males (n = 1101)				
*Sociodemographic*					
Occupational class (53y) (n,%)	5 (0.5%)				
Professional		155 (14.1)	17 (23.0)	112 (14.3)	26 (10.8)
Intermediate		458 (41.8)	34 (46.0)	325 (41.6)	99 (41.1)
Skilled (non-manual)		114 (10.4)	5 (6.8)	82 (10.5)	27 (11.2)
Skilled (manual)		269 (24.5)	15 (20.3)	191 (24.5)	63 (26.1)
Partly skilled		79 (7.2)	3 (4.1)	57 (7.3)	19 (7.9)
Unskilled		21 (1.9)	0	14 (1.8)	7 (2.9)
Educational attainment (26y) (n,%)	61 (5.5)				
No education		342 (32.9)	17 (25.0)	251 (34.0)	74 (31.8)
Less than GCE		57 (5.5)	2 (2.9)	36 (4.9)	19 (8.2)
GCE (O or A)		463 (44.5)	36 (52.9)	326 (44.1)	101 (43.4)
Degree (incl Higher)		178 (17.1)	13 (19.1)	126 (17.1)	39 (16.7)
Smoking status (53y, *current smoker*)	47 (4.3)	208 (19.7)	12 (16.7)	140 (18.6)	56 (24.4)
Leisure physical activity last wk (53y,*≥5times)*(n,%)	47 (4.3)	386 (36.6)	34 (47.2)	279 (37.1)	73 (31.7)
*Anthropometrics*					
BMI (kg/m^2^) (53y) (median (25th, 75th centile))	47 (4.3)	26.8 (24.6, 29.4)	28.1 (25.9, 29.9)	26.8 (24.5, 29.3)	26.6 (24.8, 29.3)
Height (53y) (mean, (SD))	63 (5.7)	175.5 (6.6)	178.5 (7.4)	176.0 (6.2)	172.9 (6.6)
Waist:hip ratio (53y) (mean, (SD))	47 (4.3)	0.9 (0.1)	0.9 (0.1)	0.9 (0.0)	0.9 (0.1)
*Morbidity*					
Pre-/diabetes (53y, *yes*) (n,%)	149 (13.5)	327 (34.4)	22 (33.3)	227 (33.8)	78 (36.5)
Cancer (53y, yes) (n,%)	48 (4.4)	15 (1.4)	2 (2.8)	10 (1.3)	3 (1.3)
Severe respiratory symptoms (53y, yes) (n,%)	47 (4.3)	182 (17.3)	11 (15.3)	115 (15.3)	56 (2.4)
	**Females (n = 1162)**				
*Sociodemographic*					
Occupational class (53y) (n,%)	4 (0.3)				
Professional		23 (2.0)	4 (2.1)	19 (2.3)	0
Intermediate		431 (37.2)	92 (47.9)	302 (36.4)	37 (27.2)
Skilled (non-manual)		421 (36.4)	55 (28.7)	317 (38.2)	49 (36.0)
Skilled (manual)		83 (7.2)	13 (6.8)	55 (6.6)	15 (11.0)
Partly skilled		150 (13.0)	21 (10.9)	106 (12.8)	23 (16.9)
Unskilled		50 (4.3)	7 (3.7)	31 (3.7)	12 (8.8)
Educational attainment (26y) (n,%)	64 (5.5)				
No education		336 (30.6)	39 (21.3)	244 (31.0)	53 (41.1)
Less than GCE		101 (9.2)	17 (9.3)	75 (9.5)	9 (7.0)
GCE (O or A)		595 (54.2)	110 (60.1)	421 (53.6)	64 (49.6)
Degree (incl Higher)		66 (6.0)	17 (9.3)	46 (5.9)	3 (2.3)
Smoking status (53y, *current smoker*)	21 (1.8)	216 (18.9)	29 (15.3)	152 (18.6)	35 (25.7)
Leisure physical activity last wk (53y,*≥5times*)(n,%)	21 (1.8)	416 (36.5)	80 (42.3)	309 (37.9)	27 (19.9)
*Anthropometrics*					
BMI (kg/m^2^) (53y) (median (25th, 75th centile))	28 (2.2)	26.1 (23.7, 29.7)	25.6 (23.5, 28.2)	26.0 (23.7, 29.8)	26.8 (23.8, 32.8)
Height (53y) (mean, (SD))	49 (4.2)	162.8 (6.0)	165.8 (6.6)	162.4 (5.6)	160.5 (6.1)
Waist:hip ratio (53y) (mean, (SD))	26 (2.2)	0.8 (0.1)	0.8 (0.1)	0.8 (0.1)	0.8 (0.1)
*Morbidity*					
Pre-/diabetes (53y, *yes*) (n,%)	175 (15.1)	316 (32.0)	52 (30.6)	222 (31.5)	42 (37.2)
Cancer (53y, yes) (n,%)	22 (1.9)	45 (4.0)	11 (5.8)	29 (3.6)	5 (3.7)
Severe respiratory symptoms (53y, yes) (n,%)	22 (1.9)	189 (16.6)	32 (16.9)	126 (15.5)	31 (22.8)

### Statistical analysis

Unconditional latent class trajectory models (LCTMs) [25] were developed to identify distinct groups of individuals who had similar GS trajectories. Due to observed differences in life-course GS trajectories between sexes [5], we decided a-priori to develop sex-specific trajectory models. We developed our LCTM models with the aim of first improving model fit (assessed by the Bayesian Information Criterion) and then quality of classification between classes (assessed by the entropy statistic [26]), whilst also retaining theoretical plausibility and interpretability. Entropy is a function of the posterior probabilities which each individual receives relating to how well they ‘belong’ to each class (ideally an individual has a very high probability for one class and very low probabilities for the other classes). Age was centred at visit 2 (males/females: 63.3/63.4y) to aid numerical stability. Model development considered several age functions for the trajectory shape, including linear, free-loading, quadratic, and fractional polynomials. The free-loading (males) and linear (females) models provided the best fit for the data (Tables S4 and S5). Using these, we then allowed residual variances to differ across classes and time and then we attempted to extend the models to allow residual variances to be correlated across time within each class. The best fitting models included a free-loading (males) and linear (females) function, error terms which were allowed to vary across time (but not class), and an autoregressive structure. We tested models with the number of classes ranging from 1 to 6 and the best class solution was selected based on model fit, quality of classification between classes, plausibility and interpretability of the average trajectories (Supplementary tables S4-S7). See Supplementary Text S1 for further details.

We examined the association between pre-/diabetes and GS trajectory class membership using the ‘3-step’ approach [27], which appropriately incorporates uncertainty of class assignment into regression modelling. Steps 1 and 2 refer to the process outlined above in which the growth model is estimated (step 1) and most likely class membership is assigned using the latent class posterior distribution obtained (step 2). In step 3, the most likely class is regressed (using multinomial regression) on predictor variables (i.e., ‘pre-/diabetes’), accounting for the misclassification observed in step 2. Odds ratios (ORs) and 95% confidence intervals for class membership by pre-/diabetes status (reference: ‘no pre-/diabetes’) are reported. We ran two sets of models: (i) unadjusted and (ii) adjusted for covariates listed above. Missing covariate data ranged from 0.4% (social class) to 3.2% (WHR at 53y) and were handled with 25 imputed datasets.

### Supplementary analyses

We tested the robustness of the pre-/diabetes status―GS trajectory class membership association by: (i) examining the association between pre-/diabetes status and the next best fitting GS trajectory-class solution; (ii) substituting the dichotomous ‘pre-/diabetes’ variable with continuous HbA1c and; (iii) repeating analyses using multilevel models to investigate the association between pre-/diabetes on the male- and female-specific ‘average’ trajectories.

All analyses were conducted in Mplus v.8.3 and Stata v.17.0.

## Results

1,101 (48.7%) of our sample were male (Table 1) and the prevalence of pre-/diabetes at 53y was 34.4% (males) and 32.0% (females).

### Latent GS trajectory classes

**Fig. 1:**
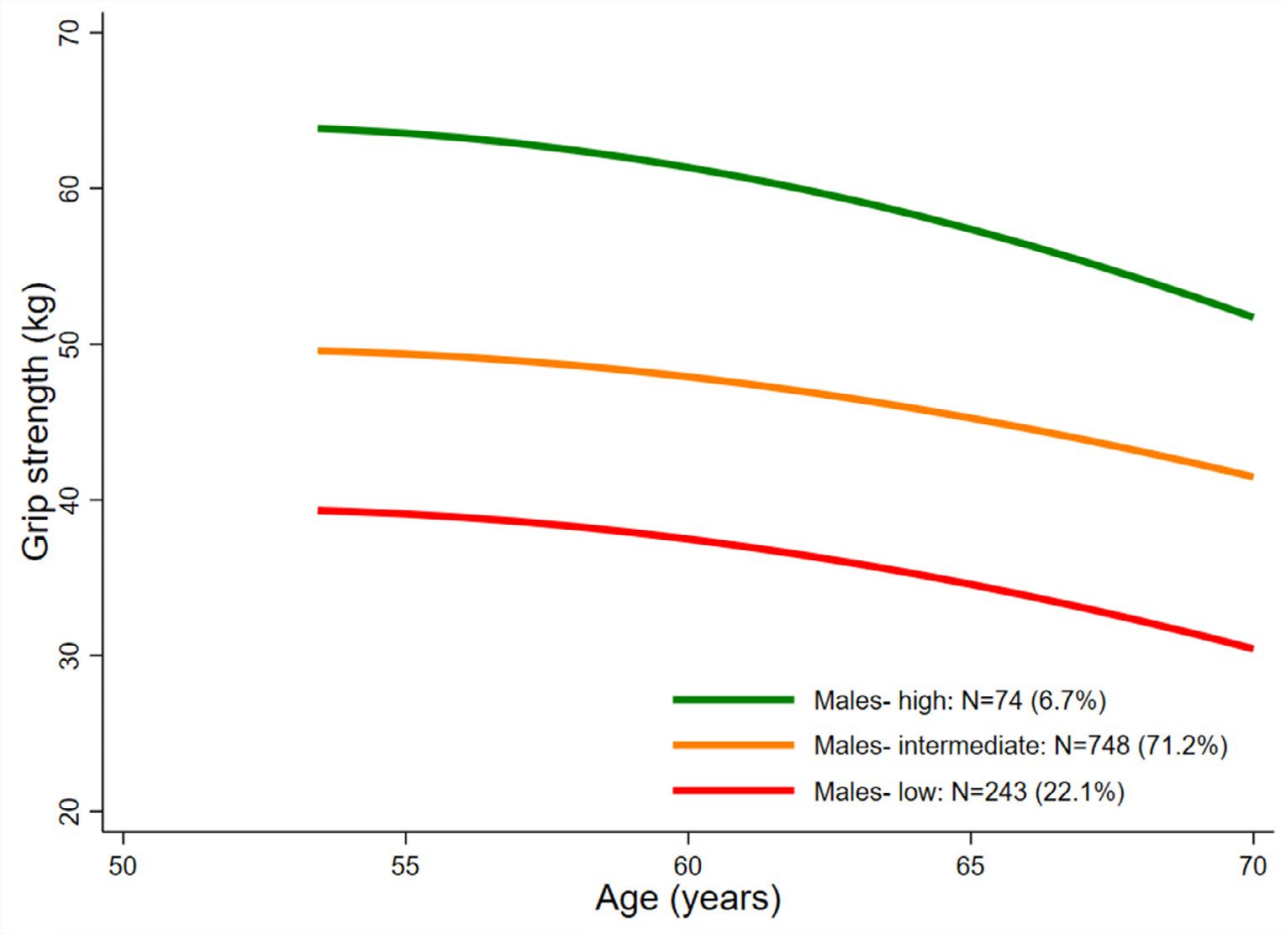
Average fitted trajectories of grip strength (kg) from the final mixture model: males

**Fig. 2:**
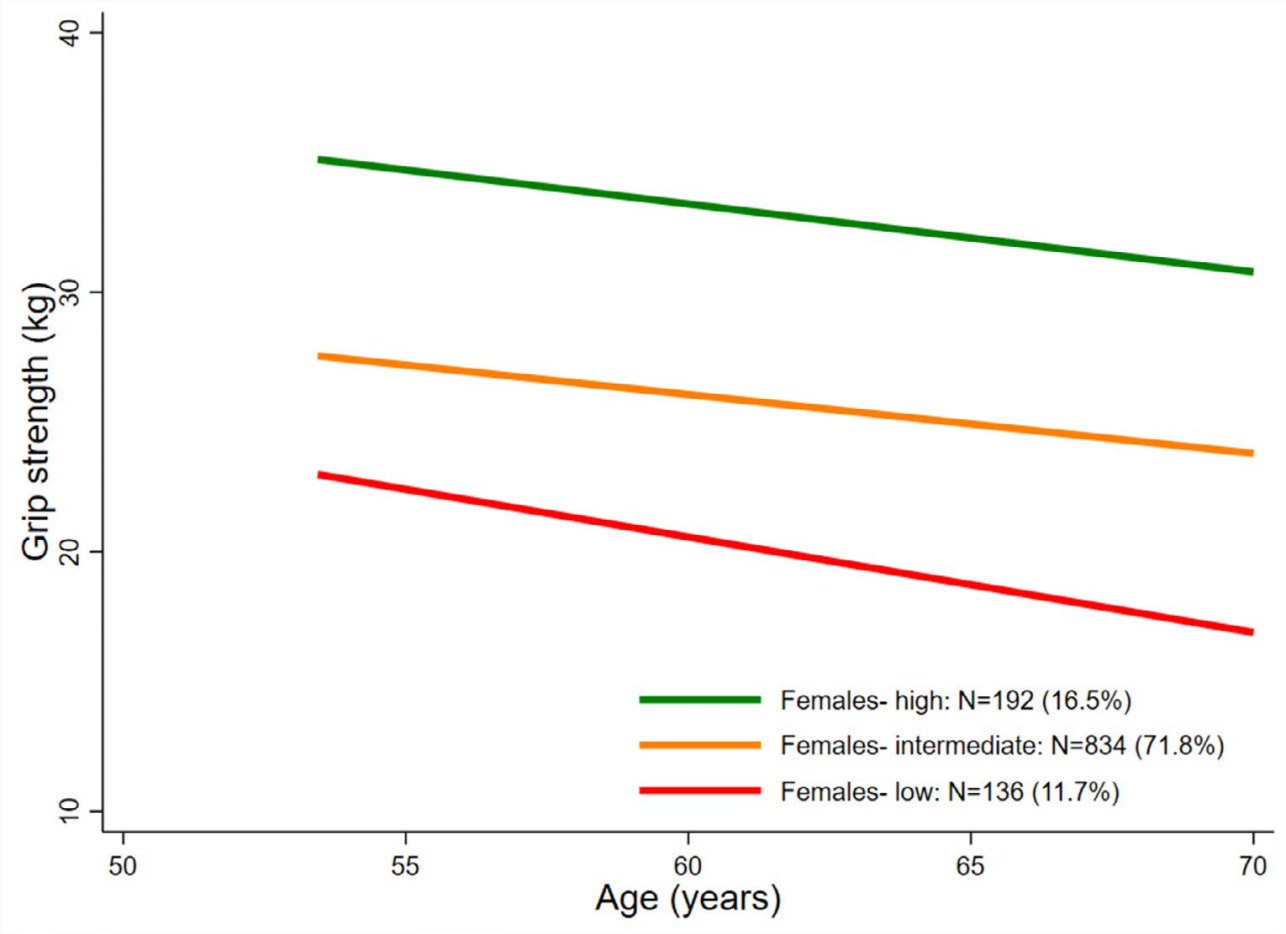
Average fitted trajectories of grip strength (kg) from the final mixture model: females

For both sexes, a LCTM with 3 classes provided the best representation of the serial GS data and the most plausible solution. Figures 1 and 2 show the average fitted trajectories for each sex. (See Supplementary Figures S2 and S3 for posterior probabilities for each of the classes and further model fit statistics in Supplementary Tables S6 and S7.)

### Class 1: ‘High’

This class comprised 6.7% of males and 16.5% of females. In males this class was characterised by a gradual decrease in average GS from ~ 64 kg at 53y to ~ 59 kg at 60-64y, followed by a more marked decrease to ~ 52 kg at 69y. In females, this class demonstrated a consistent decline in GS between 53 and 69y from ~ 35 kg to ~ 31 kg. In both sexes, average GS of this class remained the highest over the period and was thus labelled ‘High’.

### Class 2: ‘Intermediate’

This class comprised 71–72% of males and females. In males, compared to the ‘High’ class, the absolute declines in GS in this class were less marked (~ 8 kg) but the trajectory started from a lower point (~ 50 kg) at 53y. In females, while absolute declines in GS (~ 4 kg) were comparable to those observed in the ‘High’ class, this class started from a lower point (~ 28 kg) at 53y. In both sexes, the average GS of this class remained between the highest and lowest trajectories and was thus termed ‘Intermediate’.

### Class 3: ‘Low’

This class comprised 22.1% of males and 11.7% of females. In males this class exhibited a similar absolute decline in GS compared to the ‘Intermediate’ class (~ 8 kg), finishing with an average GS at 69y of ~ 31 kg. In females however, the average absolute decline in GS exhibited by this class was greater (~ 6 kg) than for the other two classes, resulting in an average GS at 69y of ~ 17 kg.

### Association between 53y pre-/diabetes status and GS trajectory membership 53-69y

There was no evidence that pre-/diabetes status was associated with class membership in either sex (Table 2). For example, ORs of being in the ‘*Low’* class (vs. ‘*High’*) for males with pre-diabetes/diabetes (vs. no-diabetes), were 1.16 (95%CI:0.57, 2.37)), reducing to 1.07 (95% CI:0.45,2.55) after adjustment. For females, corresponding ORs were 1.36 (95% CI:0.70,2.64) (unadjusted) and 0.68 (95% CI:0.25,1.84) (adjusted).

**Table 2:** Odds ratios for grip strength trajectory class membership by pre-/diabetes status

Grip strength trajectory class membership	*Class 1: High*			*Class 2: Intermediate*			*Class 3: Low*		
	N (%)	OR (95% CI)*		N (%)	OR (95% CI)*		N (%)	OR (95% CI)*	
		Model A	Model B		Model A	Model B		Model A	Model B
Males (n = 1101)									
*Pre-/diabetes*									
*Yes (n = 377)* ^*†*^	24 (6.4)	-	-	264 (70.0)	0.98 (0.47, 2.01)	0.95 (0.42, 2.11)	89 (23.6)	1.16 (0.57, 2.37)	1.07 (0.45, 2.55)
Females (n = 1162)									
*Pre-/diabetes*									
*Yes (n = 375)* ^*†*^	60 (16.0)	-	-	265 (70.6)	0.98 (0.57, 1.68)	0.99 (0.54, 1.81)	50 (13.4)	1.36 (0.70, 2.64)	0.68 (0.25, 1.84)

*OR: Odds ratios for class membership, using ‘High’ as referent outcome class and ‘no pre-/diabetes’ as reference exposure group. Model A is unadjusted; Model B is adjusted for social class (53 year), physical activity (53 year), smoking status (53 year), waist-hip ratio (53 year) and height (53 year), educational attainment (26 year), diagnoses of cancer (53 year) and ‘severe respiratory symptoms’ (53 year); ^†^after imputation (n = 25 imputations)

### Supplementary analyses

When repeating analyses using (i) the 4-class solution (Supplementary Figures S4 & S5) and (ii) substituting HbA1c as the exposure, similar findings were observed (Supplementary Tables S8 and S9). Similarly, when investigating the relationship between pre-/diabetes status and the ‘average’ GS trajectories for males and females, no association was observed in either the location (intercept term) or pattern (slope term) of the trajectory (Supplementary Figures S6 & S7).

## Discussion

Using GS data between ages 53 and 69y in a nationally representative cohort of adults in Great Britain, we observed three distinct GS trajectories in both sexes, all of which were declining. There was no evidence of association between pre-/diabetes status at 53y and membership into these trajectories.

Previous work in the NSHD grouped individuals into one of four categories of GS change between 53 and 60-64y [15, 16]. However, the availability of only two data points meant that more powerful modelling techniques could not be used to derive trajectory classes. Thus, we expand on previous work in this cohort by using a flexible data-driven approach to identify GS trajectories using data at three time points. Our finding of a declining GS trajectory between 53-69y aligns with other studies [10]. In the NSHD cohort, Dercon et al. adopted the same latent class trajectory modelling technique and identified, three distinct GS trajectories between 53-69y (‘high’, ‘reference’, ‘low’) [17]. However, as the GS trajectories were based on sex-combined analyses and modelled on a standardised scale direct comparisons between the two studies cannot be made in terms of the shape of the identified trajectories. Nonetheless, the fact that Dercon et al. also observed that the distribution of GS trajectories during this age-range could be adequately summarised into 3 non-overlapping sub-populations suggests that a basic GS trajectory profile comprising a high, intermediate and low trajectory may adequately summarise the pattern of change in GS during mid-late adulthood in the NSHD.

Our findings have important implications in terms of expected trajectories of GS in mid-late adulthood. First, by 53y there is notable variation in GS in both men and women and the observed rank order is maintained to 69y, suggesting that by this life-stage, the influence of age of onset and rate of decline are such that a single GS measure during this age-range may suffice to place an individual on their excepted trajectory. Second, we show that the rate of decline in these trajectories is not constant in males, with an apparent greater rate of decline at older ages and greater absolute declines in males in the ‘high’ class. Males also demonstrated greater absolute reductions in GS compared to females. These observations are consistent with findings that people who have greater strength at baseline are more likely to experience a faster rate of strength loss than those who are initially weaker [28].

We present novel findings regarding the pre-/diabetes status―GS trajectories association using a longitudinal design which, unlike cross-sectional studies, respected temporality. Most, but not all [29], previous cross-sectional studies demonstrated an association between diabetes status and lower GS [18, 19]. However, inferring direction of causation from such studies is challenging. Findings from longitudinal studies are equivocal. Kuh and colleagues [30] observed no association between HbA1c at 60-64y and GS at 69y in the NSHD, while Kalyani et al [20] observed an association between higher FPG at 71y and weaker average GS trajectories over seven years. In contrast, we show, in a younger sample of adults, that pre-/diabetes status was not associated with either the sex-specific sample average trajectories or the GS trajectory sub-populations.

Given known mechanisms linking excessive blood glucose and protein degradation to muscle atrophy [31–33], our finding of a lack of association between pre-/diabetes status and GS trajectory class membership over the subsequent 16 years is surprising. However, this does not preclude an association with trajectories over a longer age-range: e.g., patients with Type 2 diabetes exhibit muscle atrophy that is initially mild in middle age [34] but increases with age and duration of diabetes [35]. Thus, our period of observation may have been too early or duration of exposure to excessive blood glucose too short, to detect any relationship between pre-/diabetes status and GS trajectories. Furthermore hyperglycaemia may affect the strength/quality of muscles in the legs more than in the arms [36]. Therefore, individuals with early impairments in glucose regulation such as pre-diabetes may not yet have experienced any considerable decline in GS.

Our study has several strengths including its prospective design which enabled us to more appropriately model the relationship between pre-/diabetes status and GS trajectory classes, reducing the possibility of reverse causation. We acknowledge limitations to our study. For example, by defining pre-/diabetes status at 53y the number of individuals with excessive blood glucose levels and/or a diabetes diagnosis will have been lower than at later ages. The low prevalence of doctor-diagnosed diabetes at 53y (n = 40, 1.77%) necessitated combining this variable with an established cut-off for HbA1c [24] to identify and include pre-diabetes. Therefore, our exposure is based on a group of individuals whose glycemia was less severe than an exclusively diabetic group. Moreover, we acknowledge that the relationship between diabetes and grip strength is potentially bi-directional and likely to change over the life course [37] and future research should explore the time-varying nature of this relationship, where data permit. We accounted for several important covariates of the pre-/diabetes―GS association, although the presence of residual confounding cannot be excluded. At the outset, we aimed to develop GS trajectories using growth mixture models (GMMs). In contrast to LCTMs, GMMs allow individuals within a class to display different trajectories. Given the added complexity in developing GMMs compared to LCTMs, the relatively small number of time points (n = 3) and participants meant many of our GMMs failed to fit. While not as realistic GMMs, our LCTMs are still more likely to reflect reality than most previous studies that assume a single ‘average’ GS trajectory around which individuals vary. As in all longitudinal studies, loss to follow-up and non-response occurred, which will likely introduce selection bias and limit generalizability of our findings. Whilst no difference in average grip strength at 53y was observed between those who were included or excluded, some differences were present. For example, participants excluded from our analyses were more likely to be from a lower occupationalclass, smoke at 36y and less likely to do physical activity > 5 day/week at 43y. Furthermore, participants excluded were more likely to be classified as having pre-/diabetes and ‘severe respiratory symptoms’, suggesting we may have selected a healthier sample for analysis. Despite these limitations, the NSHD does remain generally representative of those born in Britain in the mid-20th century [38]. We also reduced the magnitude of losses to follow-up and potential bias due to missing data by using multiple imputation. Finally, although GS was measured using a different device at age 69y, it has been shown that there are no statistically significant differences in absolute values when comparing these devices [39].

## Conclusion

In both sexes, GS trajectories between 53 and 69y were best summarised with three distinct classes, namely ‘high’, ‘intermediate’ and ‘low’ trajectories. We observed no evidence of association between pre-/diabetes status at 53y and membership into these trajectory classes. Nonetheless, obtaining a comprehensive understanding of the relationship between diabetes status (and its precursor, pre-diabetes) and GS trajectories is vital to ascertain the potential contributory effect that projected increases in pre-/diabetes prevalence [40] are likely to have on the maintenance of strength and functional ability at older ages. Therefore, this work needs repeating in younger and older cohorts as the former are likely to experience a greater burden of diabetes over their life-course due to the obesity epidemic [41], while the latter are likely to have diabetes risk profiles which are currently more adverse than the cohort examined here because of age-related changes in diabetes risk.

## Electronic supplementary material

Below is the link to the electronic supplementary material.

. Supplementary Material 1. Supplementary Text S1, Tables S1-S9 and Figures S!-S7.

## Data Availability

The datasets used in this study will not be made publicly available. Access to NSHD data adheres to strict confidentiality guidelines but these data are available to bona fide researchers upon request to the NSHD Data Sharing Committee via a standard application procedure. Further details can be found at http://www.nshd.mrc.ac.uk/data; doi: 10.5522/NSHD/Q101 ; doi: 10.5522/NSHD/Q102 ; doi: 10.5522/NSHD/Q103.

## Abbreviations

FPG: Fasting plasma glucose
GMM: Growth Mixture Model
GS: Grip strength
LCTM: Latent class trajectory model
MRC: Medical Research Council
NSHD: National Survey of Health and Development
REC: Research Ethics Committee
WHR: Waist-hip ratio
