# Supplementary material for: Associations between diabetes status and grip strength trajectory sub-groups in adulthood: findings from over 16 years of follow-up in the MRC National Survey of Health and Development
Source: BMC Geriatr. 2023 Apr 4;23:213. doi: 10.1186/s12877-023-03871-9 (PMC10074704; doi:10.1186/s12877-023-03871-9)

**Associations between diabetes status and grip strength trajectory sub-groups in adulthood: findings from over 16 years of follow-up in the MRC National Survey of Health and Development**

Norris, T.^1^, Johnson, W.^2^, Cooper, R.^Ϯ3,4^ & Pinto Pereira S.M.^Ϯ*1^

^1^Institute of Sport, Exercise and Health, Division of Surgery & Interventional Science, University College London, London, United Kingdom

^2^School of Sport, Exercise and Health Sciences, Loughborough University, Loughborough, United Kingdom

^3^AGE Research Group, Translational and Clinical Research Institute, Faculty of Medical Sciences, Newcastle University, Newcastle, UK

^4^NIHR Newcastle Biomedical Research Centre, Newcastle University and Newcastle upon Tyne NHS Foundation Trust

^Ϯ^Joint senior authors

*Corresponding author

Contents

[Text S1: Latent class trajectory modelling 3](#_Toc117781746)

[Table S1 Characteristics of those included in the final sample (n=2 263) vs those excluded (n=3 099) 5](#_Toc117781747)

[Table S2. Description of the longitudinal hand grip strength (kg/10) and age data at each data collection sweep: males 6](#_Toc117781748)

[Table S3. Description of the longitudinal hand grip strength (kg/10) and age data at each data collection sweep: females 6](#_Toc117781749)

[Table S4. Comparison of the BIC between mixture models (1-6 classes) with different specifications: males 7](#_Toc117781750)

[Table S5. Comparison of the BIC between mixture models (1-6 classes) with different specifications: females 8](#_Toc117781751)

[Table S6. Summary of final latent class trajectory models (1-6 classes): males 9](#_Toc117781752)

[Table S7. Summary of final latent class trajectory models (1-6 classes): females 10](#_Toc117781753)

[Table S8. Odds ratios for class membership by pre-/diabetes status: 4 class solution 11](#_Toc117781754)

[Table S9. Odds ratios for class membership per 1% (absolute value) increase in HbA1c 12](#_Toc117781755)

[Figure S1. Proposed directed acyclic graph of the association between pre-/diabetes status and GS trajectories 13](#_Toc117781756)

[Figure S2. Distribution of posterior probabilities for assigned class membership for the selected 3-class model: males 14](#_Toc117781757)

[Figure S3. Distribution of posterior probabilities for assigned class membership for the selected 3-class model: females 15](#_Toc117781758)

[Figure S4. Average fitted trajectories of grip strength (kg) from the 4-class solution: males 16](#_Toc117781759)

[Figure S5. Average fitted trajectories of grip strength (kg) from the 4-class solution: females 17](#_Toc117781760)

[Figure S6. Mean GS trajectory curve for males with (solid line) and without (dashed line) pre-/diabetes 18](#_Toc117781761)

[Figure S7. Mean GS trajectory curve for females with (solid line) and without (dashed line) pre-/diabetes 19](#_Toc117781762)

# **Text S1: Latent class trajectory modelling**

We used Mplus (version 8.3) to develop a single latent class trajectory model that identified distinct groups of individuals who had similar adulthood grip strength (GS) trajectories between 53-69 years of age.

GS was assessed at all sweeps used in the present paper. Mplus requires the data to be in wide format, with a separate column for GS data at each sweep. As described in the methods, we dropped individuals who did not have two or more GS measurements. This reduced the sample size by 3099. Tables S2 and S3 provide a description of the sweeps and data used for analysis.

As is recommended, we developed our mixture model specification in a series of steps, with the aim to improve the Bayesian Information Criterion (BIC) and ignoring the entropy statistic, as this is not a measure of model fit(1, 2). The age scale was centred at visit 2 (age 63.3 years in males and 63.4 years in females) to aid numerical stability. For 1-6 class solutions, we fitted a series of latent class trajectory models in which the longitudinal GS response was described using each of the following age functions: linear, quadratic, free-loading and fractional polynomial. With the free-loading function, one factor loading is set to zero, another to one, and the rest are freely estimated, resulting in a type of non-linear “spline” which flexibly fits the data between adjacent time points(3). As shown in Tables S4 and S5, the free-loading and linear models (with intercept and slope variances constrained to be zero to enable convergence) provided the best fit for the data in males and females, respectively. Using these models, we next tried to relax some of the main default constraints implemented by Mplus. Firstly, we tested models that allowed the residual variances/errors to differ across classes and time, but improvements in model fit were only seen when allowing residual variances to differ across time. As recommended by Gilthorpe et al(4) we then attempted to extend the models to include a within-class autocorrelation structure for the residual variances/errors. We started with a full autoregressive correlation model (AR (1)) and after this did not converge, we fitted decreasingly complex correlation structures until converge was achieved. Final models included within class regressions of GS_t_ on GS_t-1_. This improved model fit by between 6 and 349 BIC points in males and up to 222 points in females (model 6).

This model was run for 1-6 class solutions. To avoid convergence at local minima(5), 500 random starts (for 10 iterations) were preformed, of which the best 50 models (according to log-likelihood) were run to completion (STARTS = 500 50; STITERATIONS = 10). In all instances, the best log-likelihood was replicated. A summary of the final mixture models (1-6 classes), including measures of class separation, is presented in Tables S6 and S7.

Our choice of which of the 1-6 class solutions to select for further investigation was based on, firstly, overall fit according to the BIC and, secondly, quality of classification or separation between the classes and interpretability of the average trajectories. As shown in Tables S6 and S7, the BIC reduced between k and k + 1 solutions up until a 3-class solution, after which the BIC increased.

For this reason, we now focus on discussing the choice between the 3-class and 4-class solutions. The entropy was slightly lower (i.e., worse) in the 3-class solution, while the average posterior probabilities of class membership were always greater than the proposed cut-off of 0.7 in both the 3- and 4-class solutions(6). However, in both sexes the proportion of individuals in each class with posterior probabilities>0.7 was higher in the 3-class solution compared to the 4-class. All classes in the 3-class solution comprised a reasonable number of participants, with the lowest proportion of individuals being 6.7% (males, class 1). In the 4-class solution however, the smallest proportion was <2% (males and females, class 1). We therefore selected the 3-class solution as our final model. For each sex, four figures were produced: a figure showing the distribution of posterior probabilities for assigned class membership (Figure S1 and S2) and another showing the final fitted trajectories for each class (Figures 1 and 2).

**References**

1. Van De Schoot R, Sijbrandij M, Winter SD, Depaoli S, Vermunt JK. The GRoLTS-checklist: guidelines for reporting on latent trajectory studies. Structural Equation Modeling: A Multidisciplinary Journal. 2017;24(3):451-67.

2. Schwarz G. Estimating the dimension of a model. The annals of statistics. 1978:461-4.

3. Bollen KA, Curran PJ. Latent curve models: A structural equation perspective: John Wiley & Sons; 2006.

4. Gilthorpe M, Dahly D, Tu Y-K, Kubzansky L, Goodman E. Challenges in modelling the random structure correctly in growth mixture models and the impact this has on model mixtures. Journal of developmental origins of health and disease. 2014;5(3):197-205.

5. Hipp JR, Bauer DJ. Local solutions in the estimation of growth mixture models. Psychological methods. 2006;11(1):36.

6. Nagin DS, Odgers CL. Group-based trajectory modeling in clinical research. Annual review of clinical psychology. 2010;6(1):109-38.

# **Table S1 Characteristics of those included in the final sample (n=2 263) vs those excluded (n=3 099)**

|  | **Included in final sample (n=2 263)** | | **Excluded as <2 grip strength measurements (n=3 099)** | | ***p* for diff*** |
| --- | --- | --- | --- | --- | --- |
|  | *total n* |  | *total n* |  |  |
|  |  | | | | |
| Sex (male, n, (%)) | 2 263 | 1 101 (48.7) | 3 099 | 1 714 (55.3) | <0.001 |
| Birth weight (kg, mean (SD)) |  |  |  |  |  |
| Males | 1 100 | 3.5 (0.5) | 1 698 | 3.4 (0.6) | 0.29 |
| Females | 1 158 | 3.3 (0.5) | 1 371 | 3.3 (0.5) | 0.02 |
| BMI (kg/m^2^) (11 y) |  |  |  |  |  |
| Males | 928 | 17.3 (2.1) | 1 122 | 17.3 (2.2) | 0.89 |
| Females | 973 | 17.5 (2.5) | 914 | 17.5 (2.8) | 0.74 |
| Occupational class (53y) | 2 254 |  | 1 946 |  |  |
| *Professional* |  | 178 (7.9) |  | 104 (5.3) | <0.001 |
| *Intermediate* |  | 889 (39.4) |  | 518 (26.6) |  |
| *Skilled non-manual* |  | 535 (23.7) |  | 445 (22.9) |  |
| *Skilled manual* |  | 352 (15.6) |  | 462 (23.7) |  |
| *Partly skilled manual* |  | 229 (10.2) |  | 309 (15.9) |  |
| *Unskilled manual* |  | 71 (3.2) |  |  |  |
| Smoking status (‘current’; 36y) | 2 069 | 577 (27.9) | 1 249 | 550 (44.0) | <0.001 |
| Physical activity (≥5day/wk; 43y) | 2 159 | 597 (27.7) | 1 103 | 213 (19.3) | <0.001 |
| Systolic blood pressure (mmHg) (36y) | 2 058 | 119.0 (14.2) | 1 233 | 122.1 (16.8) | <0.001 |
| Diastolic blood pressure (mmHg) (36y) | 2 056 | 76.1 (11.9) | 1 232 | 78.1 (13.2) | <0.001 |
| Pre-/diabetes (53y, *yes*) (n,%) | 1 939 | 643 (33.2) | 670 | 306 (45.7) | <0.001 |
| Cancer (53y, yes) (n,%) | 2 193 | 60 (2.7) | 791 | 33 (4.2) | 0.05 |
| Severe respiratory symptoms (53y, yes) (n,%) | 2 194 | 371 (16.9) | 791 | 197 (24.9) | <0.001 |
| Grip strength (53y) (kg, mean (SD)) |  |  |  |  |  |
| Males | 986 | 48.4 (11.8) | 292 | 47.5 (12.1) | 0.25 |
| Females | 1 052 | 28.2 (7.4) | 257 | 27.3 (7.7) | 0.11 |

*t-test for continuous & chi^2^ for categorical variables

# **Table S2. Description of the longitudinal grip strength (kg/10) and age data at each data collection sweep: males**

| **Visit** | **N** | **Age (years)** | | | | | | **Grip strength (kg/10)** | | |
| --- | --- | --- | --- | --- | --- | --- | --- | --- | --- | --- |
|  |  | **25^th^ centile** | **50^th^ centile** | **75^th^ centile** | **Min** | **Max** | **Range*** | **25^th^ centile** | **50^th^ centile** | **75^th^ centile** |
| 1 | 986 | 53.3 | 53.4 | 53.6 | 53 | 54.2 | 1.2 | 4.1 | 4.9 | 5.6 |
| 2 | 961 | 62.7 | 63.6 | 64.2 | 60.3 | 64.9 | 4.6 | 3.7 | 4.5 | 5.2 |
| 3 | 988 | 69.3 | 69.5 | 69.7 | 69 | 70.6 | 1.6 | 3.5 | 4.1 | 4.6 |

*difference between oldest and youngest participant at each sweep

# **Table S3. Description of the longitudinal grip strength (kg/10) and age data at each data collection sweep: females**

| **Visit** | **N** | **Age (years)** | | | | | | **Grip strength (kg/10)** | | |
| --- | --- | --- | --- | --- | --- | --- | --- | --- | --- | --- |
|  |  | **25^th^ centile** | **50^th^ centile** | **75^th^ centile** | **Min** | **Max** | **Range*** | **25^th^ centile** | **50^th^ centile** | **75^th^ centile** |
| 1 | 1 052 | 53.3 | 53.4 | 53.6 | 53 | 54.2 | 1.2 | 2.3 | 2.8 | 3.3 |
| 2 | 1 024 | 62.9 | 63.7 | 64.2 | 60.3 | 64.9 | 4.6 | 2.1 | 2.6 | 3.1 |
| 3 | 1 027 | 69.3 | 69.5 | 69.7 | 69 | 70.3 | 1.3 | 2.0 | 2.4 | 2.8 |

*difference between oldest and youngest participant at each sweep

# **Table S4. Comparison of the BIC between mixture models (1-6 classes) with different specifications: males**

|  | **Model 1** | **Model 2** | | **Model 3** | | **Model 4** | | **Model 5** | | **Model 6** | |
| --- | --- | --- | --- | --- | --- | --- | --- | --- | --- | --- | --- |
|  | Linear | Quadratic  polynomial | | Free loading | | Fractional polynomial (1 degree) | | Model 3 + Residual variances  (of grip strength)  allowed to differ  across classes | | Model 3 + inclusion autoregression structure^a^ | |
|  |  |  | Δ from  model 1 |  | Δ from  model 2 |  | Δ from  model 3 |  | Δ from  model 3 |  | Δ from  model 3 |
| Class |  |  |  |  |  |  |  |  |  |  |  |
| 1 | 8648 | 8645 | -3 | 8645 | 0 | 8649 | +4 | 8645 | 0 | 8296 | -349 |
| 2 | 8307 | 8289 | -18 | 8295 | +6 | 8299 | +4 | 8307 | +12 | 8187 | -108 |
| 3 | 8190 | 8175 | -15 | 8174 | -1 | 8320 | +146 | 8178 | +4 | 8142 | -32 |
| 4 | 8180 | 8164 | -16 | 8163 | -1 | 8341 | +178 | 8182 | +19 | 8147 | -16 |
| 5 | 8174 | 8163 | -11 | 8167 | +4 | 8220 | +53 | 8198 | +31 | 8157 | -10 |
| 6 | 8180 | 8176 | -4 | 8175 | -1 | 8241 | +66 | 8214 | +39 | 8169 | -6 |

^a^autoregression of adjacent grip strength measurements

# **Table S5. Comparison of the BIC between mixture models (1-6 classes) with different specifications: females**

|  | **Model 1** | **Model 2** | | **Model 3** | | **Model 4** | | **Model 5** | | **Model 6** | |
| --- | --- | --- | --- | --- | --- | --- | --- | --- | --- | --- | --- |
|  | Linear | Quadratic  polynomial | | Free loading | | Fractional polynomial (1 degree) | | Model 1 + Residual variances  (of grip strength)  allowed to differ  across classes | | Model 1 + inclusion autoregression structure^a^ | |
|  |  |  | Δ from  model 1 |  | Δ from  model 1 |  | Δ from  model 1 |  | Δ from  model 1 |  | Δ from  model 1 |
| Class |  |  |  |  |  |  |  |  |  |  |  |
| 1 | 6449 | 6454 | +5 | 6454 | 0 | 6461 | +12 | 6449 | 0 | 6227 | -222 |
| 2 | 6154 | 6150 | -4 | 6157 | +3 | 6158 | +4 | 6169 | +15 | 6126 | -28 |
| 3 | 6113 | 6123 | +10 | 6118 | +5 | 6180 | +67 | 6134 | +21 | 6110 | -3 |
| 4 | 6120 | 6130 | +10 | 6124 | +4 | 6201 | +81 | 6139 | +19 | 6122 | +2 |
| 5 | 6134 | 6138 | +4 | 6137 | +3 | 6165 | +31 | 6157 | +23 | 6134 | 0 |
| 6 | 6143 | 6155 | +12 | 6148 | +5 | 6243 | +100 | 6185 | +42 | 6148 | +5 |

^a^autoregression of adjacent grip strength measurements

# **Table S6. Summary of final latent class trajectory models* (1-6 classes): males**

| **Classes** | **1** | **2** | **3** | **4** | **5** | **6** |
| --- | --- | --- | --- | --- | --- | --- |
| AIC | 8261 | 8136 | 8077 | 8067 | 8062 | 8058 |
| BIC | 8296 | 8187 | 8142 | 8147 | 8157 | 8169 |
| BIC difference | -- | -109 | -45 | +5 | +10 | +12 |
| Entropy | -- | 0.43 | 0.64 | 0.70 | 0.62 | 0.65 |
| Posterior probability [mean] |  |  |  |  |  |  |
| Class-1 | -- | 0.81 | 0.81 | 0.73 | 0.83 | 0.83 |
| Class-2 | -- | 0.82 | 0.80 | 0.82 | 0.73 | 0.77 |
| Class-3 | -- |  | 0.84 | 0.84 | 0.77 | 0.73 |
| Class-4 | -- |  |  | 0.79 | 0.68 | 0.65 |
| Class-5 | -- |  |  |  | 0.74 | 0.72 |
| Class-6 | -- |  |  |  |  | 0.70 |
| Posterior probability > 0·7 [%] |  |  |  |  |  |  |
| Class-1 | -- | 72.5 | 70.3 | 60.0 | 78.2 | 76.8 |
| Class-2 | -- | 75.9 | 70.0 | 73.0 | 52.9 | 68.7 |
| Class-3 | -- |  | 83.0 | 81.9 | 70.6 | 57.1 |
| Class-4 | -- |  |  | 67.8 | 50.8 | 28.6 |
| Class-5 | -- |  |  |  | 55.0 | 47.1 |
| Class-6 | -- |  |  |  |  | 52.7 |
| Class membership [N (%)] |  |  |  |  |  |  |
| Class-1 | (100.0) | 484 (44.0) | 74 (6.7) | 15 (1.4) | 55 (5.0) | 56 (5.1) |
| Class-2 |  | 617 (56.0) | 243 (22.1) | 74 (6.7) | 17 (1.5) | 521 (47.3) |
| Class-3 |  |  | 784 (71.2) | 779 (70.8) | 547 (49.7) | 70 (6.4) |
| Class-4 |  |  |  | 233 (21.2) | 402 (36.5) | 14 (1.3) |
| Class-5 |  |  |  |  | 80 (7.3) | 17 (1.5) |
| Class-6 |  |  |  |  |  | 423 (38.4) |

*Free-loading age function with heteroscedastic errors (across time but not class), and a first-order autoregressive structure (AR1) to model autocorrelation

# **Table S7. Summary of final latent class trajectory models* (1-6 classes): females**

| **Classes** | **1** | **2** | **3** | **4** | **5** | **6** |
| --- | --- | --- | --- | --- | --- | --- |
| AIC | 6197 | 6081 | 6050 | 6046 | 6043 | 6042 |
| BIC | 6227 | 6126 | 6110 | 6122 | 6134 | 6148 |
| BIC difference | -- | -101 | -16 | +12 | +12 | +14 |
| Entropy | -- | 0.49 | 0.58 | 0.61 | 0.65 | 0.65 |
| Posterior probability [mean] |  |  |  |  |  |  |
| Class-1 | -- | 0.86 | 0.74 | 0.74 | 0.74 | 0.74 |
| Class-2 | -- | 0.80 | 0.82 | 0.76 | 0.79 | 0.74 |
| Class-3 | -- |  | 0.79 | 0.74 | 0.78 | 0.65 |
| Class-4 | -- |  |  | 0.79 | 0.73 | 0.75 |
| Class-5 | -- |  |  |  | 0.76 | 0.77 |
| Class-6 | -- |  |  |  |  | 0.71 |
| Posterior probability > 0·7 [%] |  |  |  |  |  |  |
| Class-1 | -- | 81.9 | 54.4 | 45.5 | 50.0 | 47.4 |
| Class-2 | -- | 71.0 | 81.2 | 60.0 | 75.0 | 63.2 |
| Class-3 | -- |  | 66.2 | 62.1 | 73.1 | 37.5 |
| Class-4 | -- |  |  | 74.1 | 59.6 | 63.3 |
| Class-5 | -- |  |  |  | 58.4 | 63.2 |
| Class-6 | -- |  |  |  |  | 55.2 |
| Class membership [N (%)] |  |  |  |  |  |  |
| Class-1 | (100.00) | 827 (71.2) | 136 (11.7) | 22 (1.9) | 30 (2.6) | 19 (1.6) |
| Class-2 |  | 335 (28.8) | 834 (71.8) | 75 (6.5) | 4 (0.3) | 668 (57.5) |
| Class-3 |  |  | 192 (16.5) | 301 (25.9) | 737 (63.4) | 8 (0.7) |
| Class-4 |  |  |  | 764 (65.7) | 314 (27.0) | 218 (18.8) |
| Class-5 |  |  |  |  | 77 (6.6) | 19 (1.6) |
| Class-6 |  |  |  |  |  | 230 (19.8) |

*Linear age function with heteroscedastic errors (across time but not class), and a first-order autoregressive structure (AR1) to model autocorrelation

# **Table S8. Odds ratios for grip strength trajectory class membership by pre-/diabetes status: 4 class solution**

| **Grip strength trajectory class membership** | *Class 1: High* | | *Class 2: Upper-middle* | | *Class 3: Lower-middle* | | *Class 4: Low* | |
| --- | --- | --- | --- | --- | --- | --- | --- | --- |
|  | OR (95% CI)* | | OR (95% CI)* | | OR (95% CI)* | | OR (95% CI)* | |
|  | Model A | Model B | Model A | Model B | Model A | Model B | Model A | Model B |
| Males (n=1101) |  | |  | |  | |  | |
| *Pre-/diabetes* |  | |  | |  | |  | |
| *Yes (n=377)^Ϯ^* | - | - | 0.91  (0.45, 1.83) | 0.89  (0.41, 1.93) | 1.08  (0.53, 2.18) | 1.03  (0.42, 2.54) | 0.67  (0.11, 4.16) | 0.52  (0.05,5.25) |
|  |  | |  | |  | |  | |
| Females (n=1162) |  | |  | |  | |  | |
| *Pre-/diabetes* |  | |  | |  | |  | |
| *Yes (n=375) ^Ϯ^* | - | - | 0.35  (0.08, 1.55) | 0.22  (0.01, 8.22) | 0.36  (0.11, 1.25) | 0.22  (0.01, 6.96) | 0.58  (0.14, 2.43) | 0.17 (0.01,6.01) |

*OR: Odds ratios for class membership, using ‘High’ as referent outcome class and ‘no pre-/diabetes’ as reference exposure group. Model A is unadjusted; Model B is adjusted for social class (53yr), physical activity (53yr), smoking status (53yr), waist-hip ratio (53yr) and height (53yr), educational attainment (26yr), diagnoses of cancer (53yr) and ‘severe respiratory symptoms’ (53yr); *^Ϯ^*after imputation (n=25 imputations)

# **Table S9. Odds ratios for grip strength trajectory class membership per 1% (absolute value) increase in HbA1c**

| **Grip strength trajectory class membership** | *Class 1: Stable high* | | *Class 2: Intermediate* | | *Class 3: Stable low* | |
| --- | --- | --- | --- | --- | --- | --- |
|  | OR (95% CI)* | | OR (95% CI)* | | OR (95% CI)* | |
|  | Model A | Model B | Model A | Model B | Model A | Model B |
| Males (n=1101) |  | |  | |  | |
| *HbA1c (%)* | - | - | 1.17 (0.67, 2.06) | 1.14 (0.63, 2.06) | 1.26 (0.69, 2.27) | 1.24 (0.61, 2.52) |
|  |  | |  | |  | |
| Females (n=1162) |  | |  | |  | |
| *HbA1c (%)* | - | - | 1.00 (0.51, 1.95) | 1.04 (0.64, 1.71) | 1.79 (0.77, 4.18) | 0.95 (0.46, 1.98) |

*OR: Odds ratios for class membership, using ‘High’ as referent outcome class, per unit increase in HbA1c. Model A is unadjusted; Model B is adjusted for social class (53yr), physical activity (53yr), smoking status (53yr), waist-hip ratio (53yr) and height (53yr), educational attainment (26yr), diagnoses of cancer (53yr) and 'severe respiratory symptoms’ (53yr); imputed with n=25 imputations

# **Figure S1. Proposed directed acyclic graph of the association between pre-/diabetes status and grip strength trajectories**


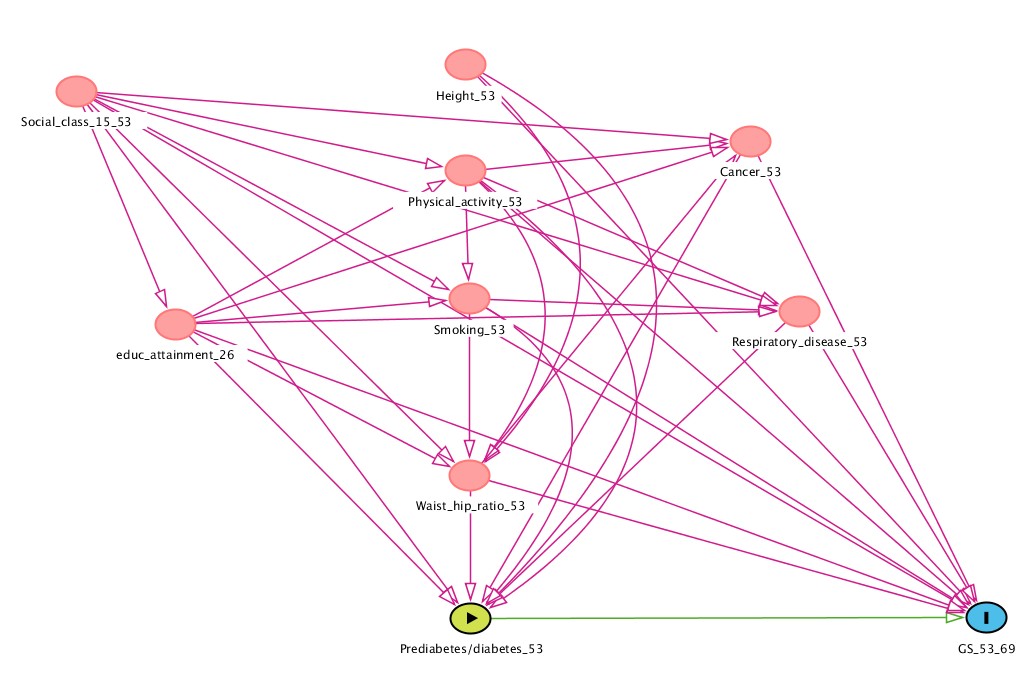


# **Figure S2. Distribution of posterior probabilities for assigned class membership for the selected 3-class model: males**


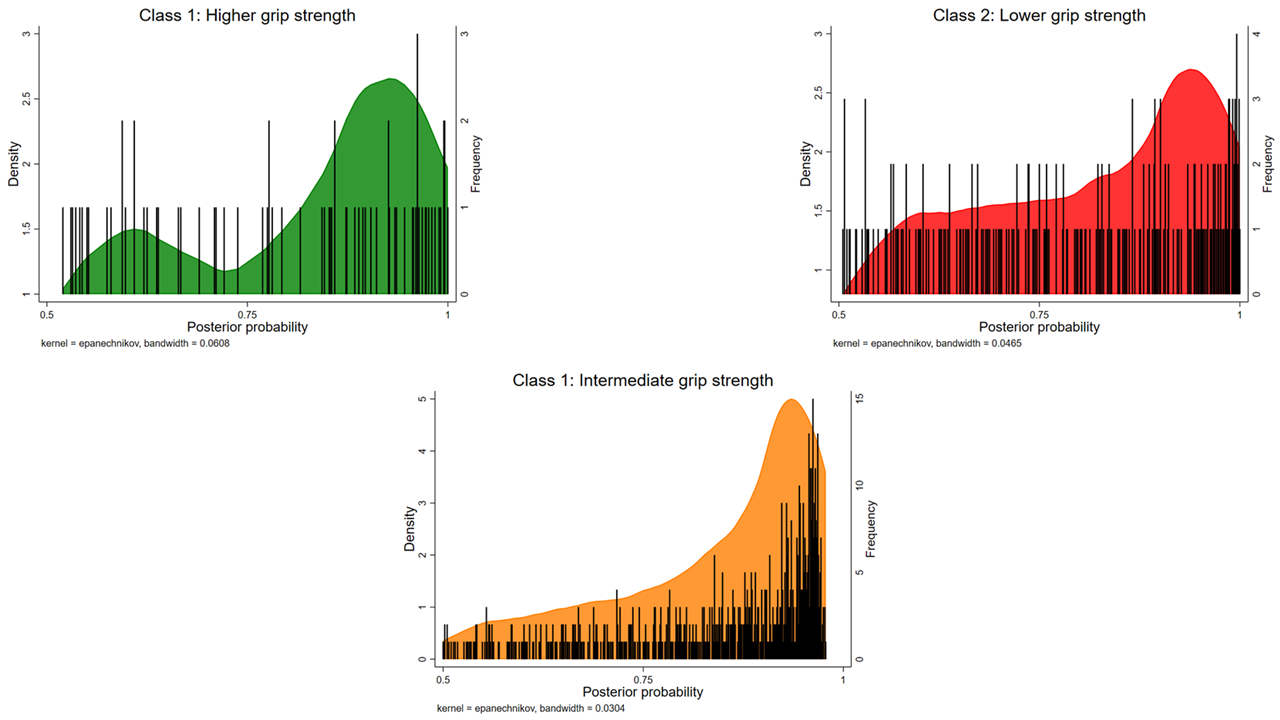


# **Figure S3. Distribution of posterior probabilities for assigned class membership for the selected 3-class model: females**


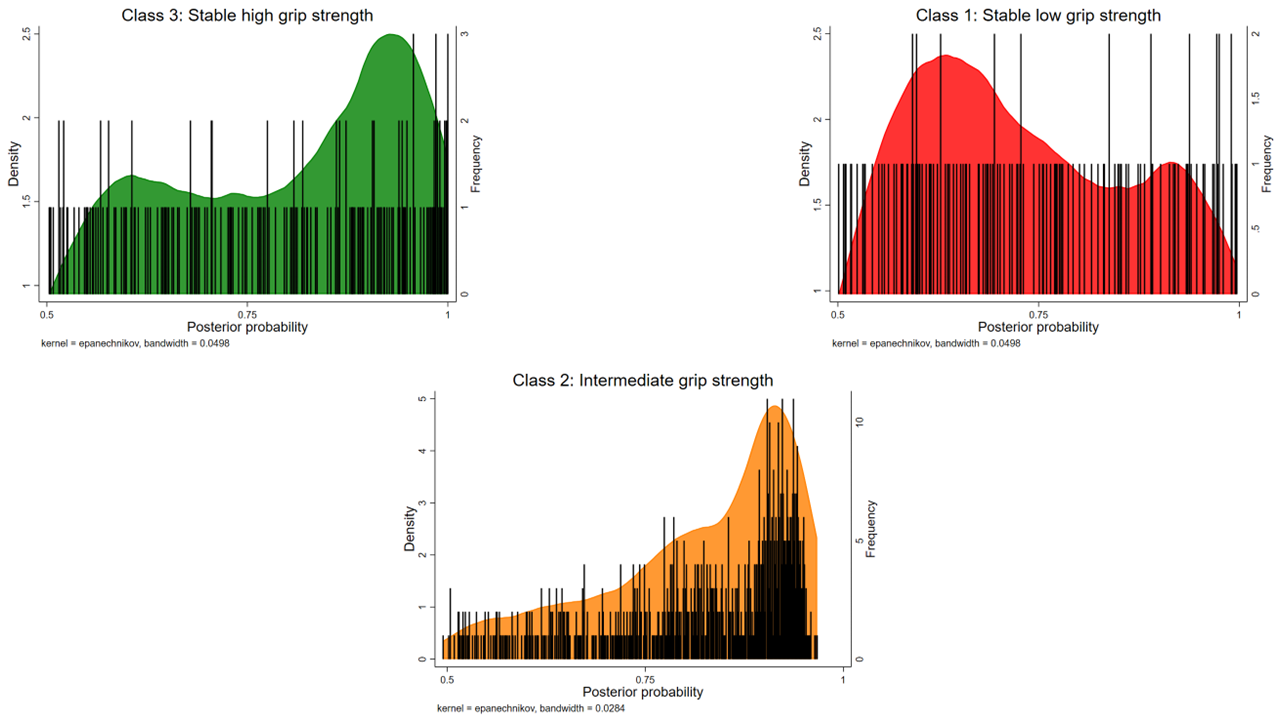


# **Figure S4. Average fitted trajectories of grip strength (kg) from the 4-class solution: males**


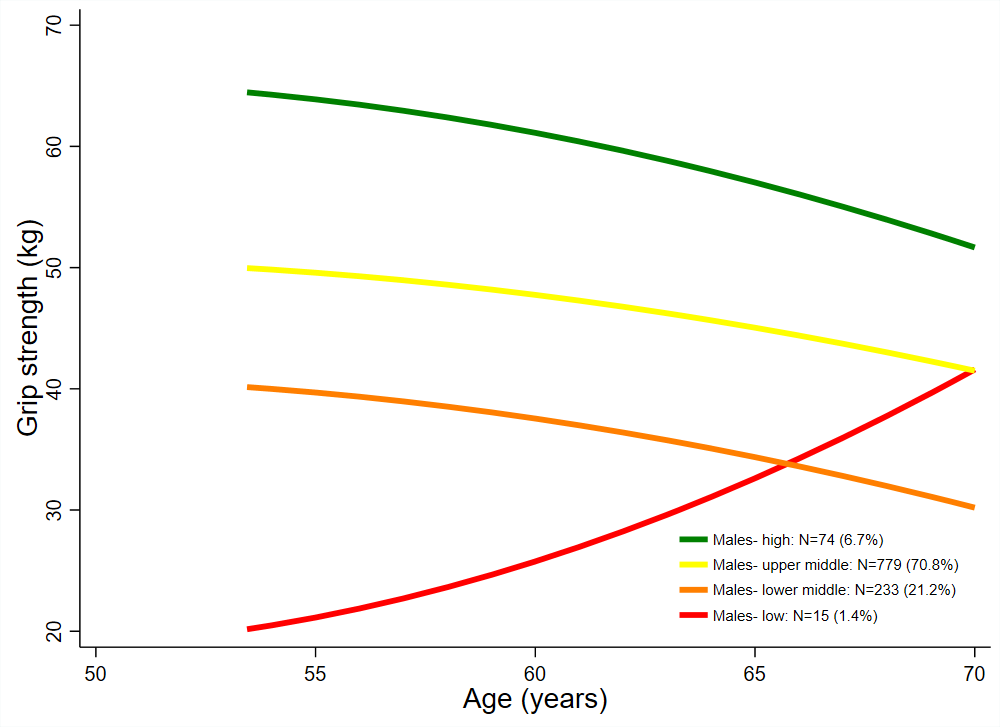


# **Figure S5. Average fitted trajectories of grip strength (kg) from the 4-class solution: females**


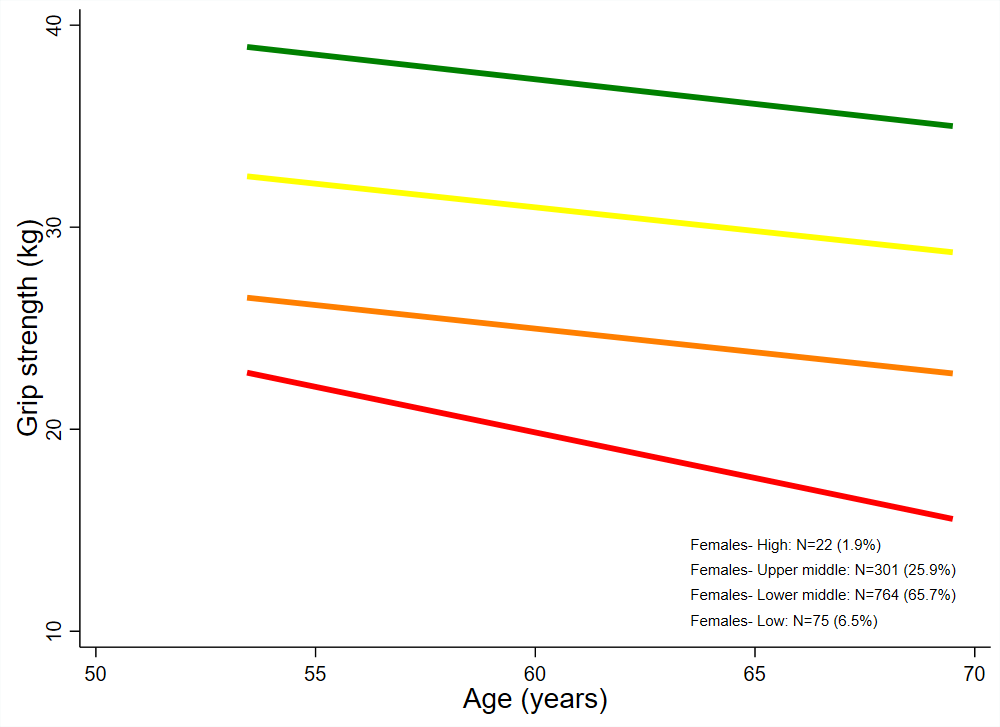


# **Figure S6. Mean grip strength (GS) trajectory curve for males with (solid line) and without (dashed line) pre-/diabetes from multi-level model**


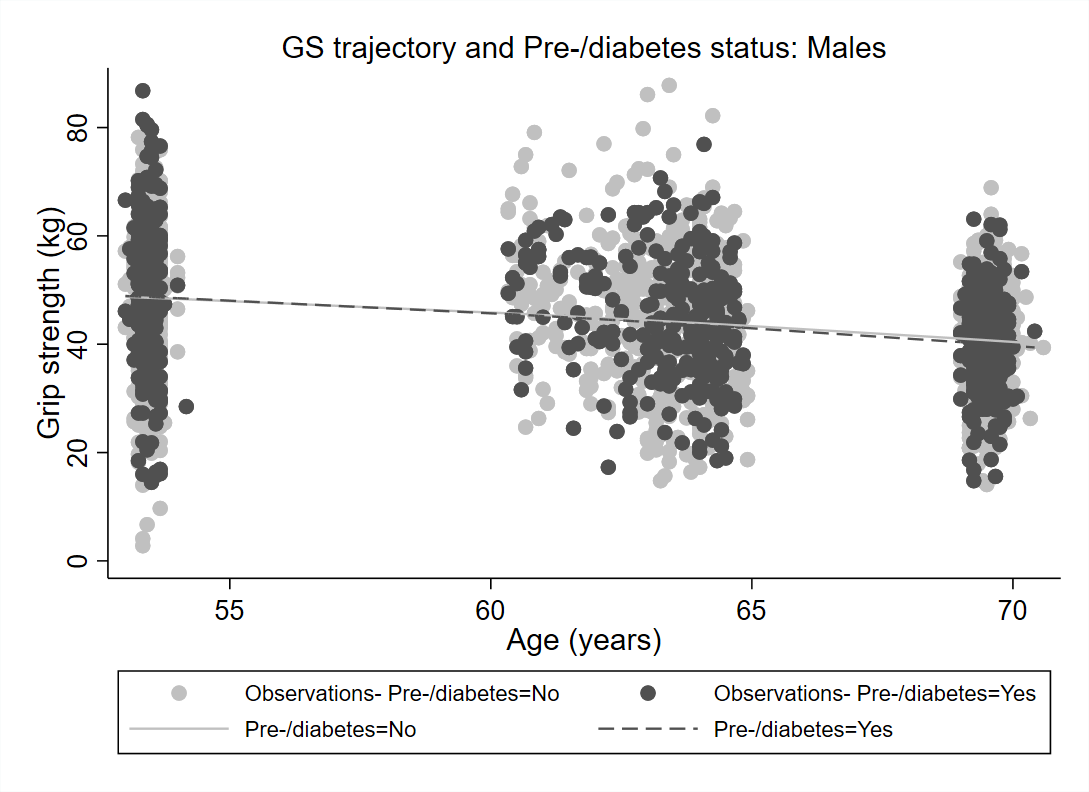


# **Figure S7. Mean grip strength (GS) trajectory curve for females with (solid line) and without (dashed line) pre-/diabetes from multi-level model**


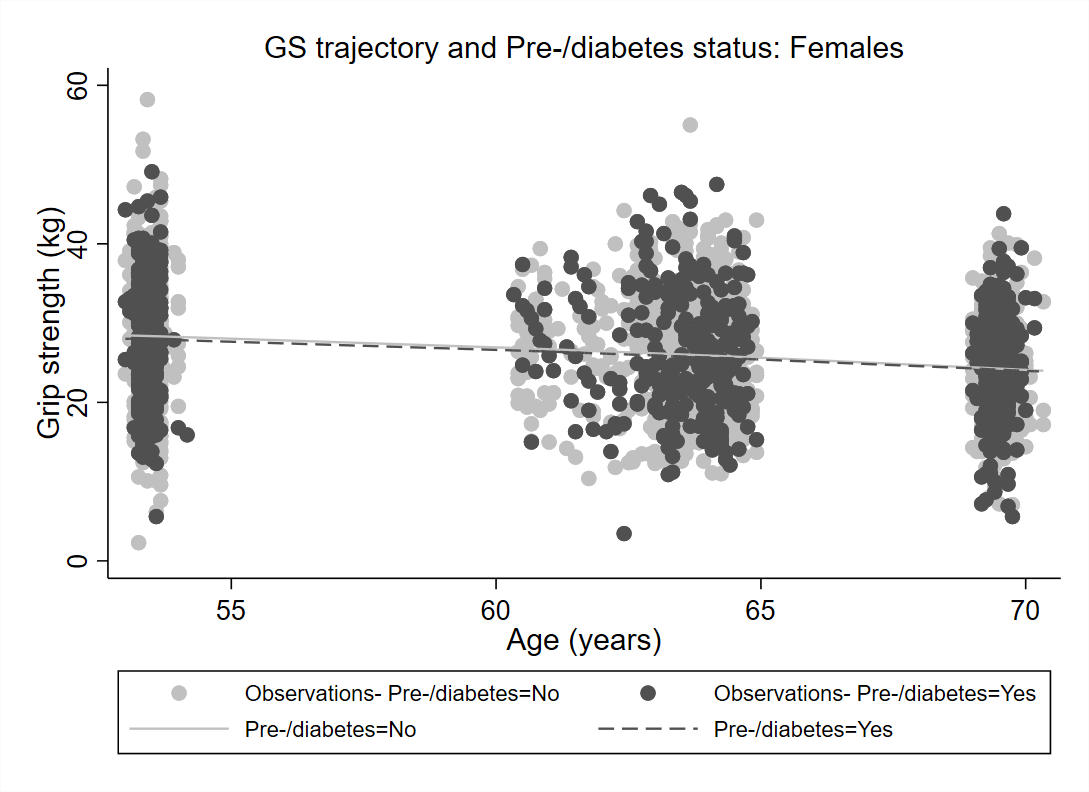

Supplement: Supplementary file 1 — . Supplementary Material 1. Supplementary Text S1, Tables S1-S9 and Figures S!-S7. [file 12877_2023_3871_MOESM1_ESM.docx]
